# The Kinematic Models of the SINS and Its Errors on the SE(3) Group in the Earth-Centered Inertial Coordinate System

**DOI:** 10.3390/s24123864

**Published:** 2024-06-14

**Authors:** Ke Fang, Tijing Cai, Bo Wang

**Affiliations:** 1 School of Instrument Science and Engineering, Southeast University, Nanjing 210096, China; fangke@seu.edu.cn; 2 Nanjing Research Institute of Electronics Engineering, Nanjing 210007, China; wangbo@seu.edu.cn

**Keywords:** attitude estimation, common frame errors, error separation and decoupling, Lie group, SINS kinematics

## Abstract

In this paper, the kinematic models of the Strapdown Inertial Navigation System (SINS) and its errors on the SE(3) group in the Earth-Centered Inertial frame (ECI) are established. On the one hand, with the ECI frame being regarded as the reference, based on the joint representation of attitude and velocity on the SE(3) group, the dynamic of the local geographic coordinate system (*n*-frame) and the body coordinate system (*b*-frame) evolve on the differentiable manifold, respectively, and the high-order expansion of the Baker–Campbell–Haussdorff equation compensates for the non-commutative motion errors stimulated by strong maneuverability. On the other hand, the kinematics of the left- and right-invariant errors of the *n*-frame and the *b*-frame on the SE(3) group are separately derived, where the errors of the *b*-frame completely depend on inertial sensor errors, while the errors of the *n*-frame rely on position errors and velocity errors. In this way, the errors brought by the inconsistency of the reference coordinate system are tackled, and a novel attitude error definition is introduced to separate and decouple the factors affecting the dynamic of the *n*-frame errors and the *b*-frame errors for better attitude estimation. Through a turntable experiment and a car-mounted field experiment, the effectiveness of the proposed kinematic models in estimating attitude has been verified, with a remarkable improvement in yaw angle accuracy in the case of large initial misalignment angles, and the models developed have better robustness compared to the traditional SE(3) group-based model.

## 1. Introduction

The Strapdown Inertial Navigation System (SINS) is an autonomous navigation system independent of external information, and is one of the primary navigation means for aircrafts, vessels, cars, missiles, unmanned vehicles, and smart wearable devices [1,2]. However, its navigation errors tend to diverge over time, necessitating integration with other navigation systems such as the Global Navigation Satellite System (GNSS), Odometer and the Doppler Velocity Log (DVL) using the Kalman Filter (KF) [3]. Attitude estimation, which involves determining the orientation of the virtual platform (or the body coordinate system, denoted as *b*-frame) relative to the local geographic coordinate system (denoted as *n*-frame), has been a critical challenge that needs to be solved in the strapdown inertial navigation and filtering algorithms [4].

In conventional applications, the consistency between the body’s angular and linear motion is disregarded, i.e., the attitude and its error are represented in the special orthogonal group SO(3), whereas the velocity, position, and their errors are located in Euclidean space. This incongruence deteriorates the navigation and filtering precision, causing the so-called “common frame” errors [5,6]. Fortunately, the three-dimensional motion description based on the special Euclidean group SE(3) provides a rational co-representation of attitude and velocity, allowing body motion and its errors to evolve on the same nonlinear differentiable manifold [7,8]. Wu et al. addressed the discrepancy between rotation and translation and simultaneously described angular and linear motion using dual quaternion kinematic equations [9]. Mao et al. derived a SINS updating method on the SE(3) group and the method has the same accuracy as the dual quaternion-based model [10]. In their work, Mao provided a joint representation of attitude and velocity, and the common frame error during the SINS update process can be eliminated through the expansion of the Baker–Campbell–Haussdorff (BCH) equation. As for the resolution of the common frame error in the context of SINS error propagation, Barrau and Bonnabel proposed the Invariant Extended Kalman Filter (IEKF), which possesses a notable feature where the error propagation is independent of the actual trajectories if the group affine property is satisfied [11,12]. Chang et al. constructed left- and right-invariant error models of the transformed INS mechanization on the ${\mathrm{SE}}_{2}\left(3\right)$ group in the Earth-Centered Earth-Fixed (ECEF) coordinate system (denoted as *e*-frame) to address the problem of the group affine property not being met when the *n*-frame is selected as the reference navigation coordinate system and the Earth’s rotation and the Coriolis effect cannot be neglected, and analyzed their performance in the SINS/GNSS and SINS/Odometer integrated systems [13]. Zhu et al. derived the quaternion-based error model on the SE(3) group and compared it to the Euler angle error model and its counterpart on the SO(3) group [14]. Qian et al. examined the necessity of the Lie group error model from three perspectives: the stability of variance, estimation accuracy, and observability, and proved its superiority to traditional error models [15].

The attitude matrix used to depict the orientation of the vehicles varies depending on the reference coordinate system, resulting in differences in the definition of the attitude error and the corresponding error kinematics. Therefore, another noteworthy consideration is the definition of the attitude error, which is the deviation between the true attitude matrix $C_{b}^{n}$ and the computational attitude matrix ${\hat{C}}_{b}^{n}$. The most widely accepted definition is to assume that the computational body coordinate system ($b^{\prime }$-frame) coincides with the *b*-frame and to define the attitude error as the difference between the true geographic coordinate system and the computational geographic coordinate system ($n^{\prime }$-frame). Traditionally, both Euler angle and quaternion error models correspond to the attitude error matrix $C_{n^{\prime }}^{n}$. Thinking from another perspective, Creamer and Suh put forward the definition of attitude error of the *b*-frame, i.e., $C_{b^{\prime }}^{b}$, however, no exhaustive analysis on the topic has been done [16,17]. Zhu et al. conducted research on the multiplicative quaternion model and additive quaternion model for the attitude error of the *n*-frame and the *b*-frame, respectively, and indicated that the attitude error model of the *b*-frame is more appropriate for the situations with large initial misalignment angles [18].

While extensive research has been conducted on Lie group-based mathematical models of SINS, limitations remain. On the one hand, the relative attitude representation between two moving coordinate systems. i.e., the *b*-frame and the *n*-frame, couples the factors that affect their orientation errors due to the introduction of the Coriolis effect, and hence, the derived Lie group model cannot meet the global state independence characteristics. On the other hand, these studies have not fully explored the inherent relationship between the Lie group-based SINS models and attitude representations. Motivated by these considerations, the authors consider that the inertial coordinate system, as a quasi-stationary coordinate system, is an inherent reference for defining attitude and its error. Therefore, a novel attitude definition method referred to the inertial coordinate system is proposed, which considers the *b*-frame attitude error and the *n*-frame attitude error separately. Specifically, based on the algorithm framework of the SE(3) group, the error model on the SE(3) group is constructed based on the SINS update algorithm, and the error kinematics equation is deduced. First, the kinematic equations of the *n*-frame and the *b*-frame with respect to the Earth-Centered Inertial (ECI) frame (denoted as $i_{0}$-frame) are derived, and in this way, the dynamics of the *b*-frame is completely determined by the information provided by inertial sensors, while the dynamics of the *n*-frame relies on the computational position and velocity. Based on the aforementioned SINS kinematics, the errors of the *n*-frame and the *b*-frame relative to the $i_{0}$-frame are also expressed in the form of SE(3) group elements, and their kinematic models are constructed. Since the $i_{0}$-frame is assumed to be absolutely accurate and quasi-static, the kinematics of the *n*-frame errors and the *b*-frame errors are separated as two independent processes and individually evolved on the differentiable manifold, so as to decouple the error terms affecting them and acquire better attitude estimation. In this way, the navigation parameters and error parameters of SINS are all included in the same SE(3) group space, and its update iteration and error propagation can be completely achieved through the same Lie group algorithm. Moreover, the error propagation of the *b*-frame is globally independent, while the error propagation of the *n*-frame is not. Fortunately, this will not affect practical applications, because of the integrated effect of complementary navigation systems. However, the rapid correction on large initial misalignment angles is particularly noteworthy.

The remainder of this paper is organized as follows. The SINS kinematics on the SE(3) group in the $i_{0}$-frame is derived in Section 2. Section 3 deduces the corresponding left- and right-invariant error models on the SE(3) group and their kinematics and briefly introduces the associated filtering model. Section 4 verifies the effectiveness of the proposed models through a turntable experiment and a car-mounted field experiment. The conclusion and possible future work are given in Section 5.

## 2. The SINS Kinematics on the SE(3) Group in the $i_{0}$-Frame

According to Newton’s second law, in an inertial coordinate system (*i*-frame), the body’s absolute acceleration is the sum of the gravity acceleration and the specific force acceleration, that is

(1) $${\dot{v}}^{i}=f^{i}+g^{i}$$

where $f^{i}$ is the specific force acceleration in the *i*-frame, $g^{i}$ is the gravity acceleration in the *i*-frame, and $v^{i}$ is the velocity in the *i*-frame.

For the motion of a rigid body on Earth, a SINS accelerometer outputs a specific force, and a gyroscope provides the angular motion information of the *b*-frame relative to the *i*-frame. Thus, the attitude matrix $C_{b}^{i}$ and the velocity increment $v_{f}^{i}$ caused by the specific force (referred as specific force velocity) can be determined.

Selecting the $i_{0}$-frame as the reference inertial coordinate system, $C_{b}^{i_{0}}$ and $v_{f}^{i_{0}}$ are combined to form an SE(3) group element, i.e.,

(2) $$T_{b}^{i_{0}}=\left[\begin{array}{cc} C_{b}^{i_{0}} & v_{f}^{i_{0}}\\ 0_{1\times 3} & 1 \end{array}\right]\in \mathrm{SE}\left(3\right)$$

Taking the derivative of (2), the kinematic equation of the *b*-frame can be acquired.

(3) $$\begin{array}{cc} {\dot{T}}_{b}^{i_{0}} & =\left[\begin{array}{cc} {\dot{C}}_{b}^{i_{0}} & {\dot{v}}_{f}^{i_{0}}\\ 0_{1\times 3} & 0 \end{array}\right]=\left[\begin{array}{cc} C_{b}^{i_{0}}\left(\omega_{i_{0}b}^{b}\times \right) & f^{i_{0}}\\ 0_{1\times 3} & 0 \end{array}\right]\\ \; & =\left[\begin{array}{cc} C_{b}^{i_{0}} & v_{f}^{i_{0}}\\ 0_{1\times 3} & 1 \end{array}\right]\left[\begin{array}{cc} \left(\omega_{i_{0}b}^{b}\times \right) & f^{b}\\ 0_{1\times 3} & 0 \end{array}\right]≜T_{b}^{i_{0}}\Xi_{b}^{i_{0}} \end{array}$$

where $\omega_{i_{0}b}^{b}$ is the projection of the angular velocity of the *b*-frame relative to the $i_{0}$-frame in the *b*-frame, measured by the gyroscope, and $f^{b}$ is the specific force in the *b*-frame, measured by the accelerometer. $\Xi_{b}^{i_{0}}$ is the related Lie algebra to $T_{b}^{i_{0}}$.

Likewise, the attitude matrix $C_{n}^{i_{0}}$ and the velocity increment $v_{g}^{i_{0}}$ generated by the gravity acceleration (referred to as gravity velocity) form an SE(3) group element as well.

(4) $$T_{n}^{i_{0}}=\left[\begin{array}{cc} C_{n}^{i_{0}} & v_{g}^{i_{0}}\\ 0_{1\times 3} & 1 \end{array}\right]\in \mathrm{SE}\left(3\right)$$

Taking the derivative of (4), the kinematic equation of the *n*-frame can be acquired.

(5) $$\begin{array}{cc} {\dot{T}}_{n}^{i_{0}} & =\left[\begin{array}{cc} {\dot{C}}_{n}^{i_{0}} & {\dot{v}}_{g}^{i_{0}}\\ 0_{1\times 3} & 0 \end{array}\right]=\left[\begin{array}{cc} C_{n}^{i_{0}}\left(\omega_{i_{0}n}^{n}\times \right) & g^{i_{0}}\\ 0_{1\times 3} & 0 \end{array}\right]\\ \; & =\left[\begin{array}{cc} C_{n}^{i_{0}} & v_{g}^{i_{0}}\\ 0_{1\times 3} & 1 \end{array}\right]\left[\begin{array}{cc} \left(\omega_{i_{0}n}^{n}\times \right) & g^{n}\\ 0_{1\times 3} & 0 \end{array}\right]≜T_{n}^{i_{0}}\Xi_{n}^{i_{0}} \end{array}$$

where $\omega_{i_{0}n}^{n}$ is the projection of the angular velocity of the *n*-frame relative to the $i_{0}$-frame in the *n*-frame, which can be calculated by (6). $g^{n}$ is the local gravity vector in the *n*-frame, which can be determined by the position and gravity formulas. $\Xi_{n}^{i_{0}}$ is the related Lie algebra to $T_{n}^{i_{0}}$.

(6) $$\omega_{i_{0}n}^{n}=\left[\begin{array}{c} -\frac{v_{N}}{R_{M}+h}\\ \omega_{ie}\cos L+\frac{v_{E}}{R_{N}+h}\\ \omega_{ie}\sin L+\frac{v_{E}\tan L}{R_{N}+h} \end{array}\right]$$

where $\omega_{ie}$ is the norm of the Earth’s angular velocity. $v_{E}$ and $v_{N}$ are the eastward and northward velocity in the *n*-frame, respectively. *L* is the geocentric latitude and *h* is the altitude. $R_{M}$ and $R_{N}$ are the meridian radius and prime vertical radius of the Earth, respectively.

However, due to the Coriolis effect, the compensation for the Coriolis acceleration needs to be considered when transforming the velocity in the $i_{0}$-frame to the rotating *n*-frame, that is

(7) $${\dot{v}}_{corr}^{n}=-\left(\omega_{i_{0}e}^{n}+\omega_{i_{0}n}^{n}\right)\times v^{n}$$

where ${\dot{v}}_{corr}^{n}$ is the Coriolis acceleration, $\omega_{i_{0}e}^{n}$ is the projection of the Earth’s angular velocity in the *n*-frame, which is calculated by (8), and $v^{n}$ is the velocity vector in the *n*-frame.

(8) $$\omega_{i_{0}e}^{n}={\left[\begin{array}{ccc} 0 & \omega_{ie}\cos L & \omega_{ie}\sin L \end{array}\right]}^{T}$$

**Remark** **1.** *The solution of the SE(3) group differential equations as shown in (3) and (5) is*

(9) $$T\left(t\right)=T\left(0\right)\exp\left[\xi \left(t\right)\right]$$

*where the relationship between $\xi \left(t\right)$ and $\Xi \left(t\right)$ can be obtained by the BCH equation shown in (10), thus the kinematic equations shown in (3) and (5) can be solved.*

(10) $$\dot{\xi }\left(t\right)=\Xi \left(t\right)+\frac{1}{2}\left[\xi \left(t\right),\Xi \left(t\right)\right]+\frac{1}{12}\left[\xi \left(t\right),\left[\xi \left(t\right),\Xi \left(t\right)\right]\right]+\cdots$$

*where* $\left[\cdot \right]$ *is the Lie Bracket operation, and the coefficients of each term form the Bernoulli series.*

Calculating the second term of (10) and (11) can be derived.

(11) $$\xi_{2}=\left[\begin{array}{cc} \left[\frac{1}{2}\int_{t}^{t+\Delta t}\left(\Delta \theta \times \omega \right)\times dt\right] & \frac{1}{2}\int_{t}^{t+\Delta t}\left(\Delta \theta \times a+\Delta v\times \omega \right)dt\\ 0_{1\times 3} & 0 \end{array}\right]$$

where $\xi_{2}$ is the second term of (10). Δθ is the angular increment generated by the angular motion, and ω is the angular velocity. Δv is the velocity increment caused by the linear motion, and a is the corresponding acceleration.

It can be found that $\frac{1}{2}\int_{t}^{t+\Delta t}\left(\Delta \theta \times \omega \right)dt$ is exactly the cone error compensation term and $\frac{1}{2}\int_{t}^{t+\Delta t}\left(\Delta \theta \times a+\Delta v\times \omega \right)dt$ is the sculling error compensation term in conventional multi-sample algorithms. When applying a high-order expansion to (10), the non-commutative error generated by the high maneuverability can be compensated without lowering the updating frequency like the multi-sample algorithm.

## 3. The Error Kinematics on the SE(3) Group in the $i_{0}$-Frame

### 3.1. Left-Invariant Error Definition and Kinematics

According to the Lie group invariant theory [11,19], the left-invariant error definition of $T_{b}^{i_{0}}$ is

(12) $$\begin{array}{cc} \delta_{l}^{i_{0}b} & ={\left({\hat{T}}_{b}^{i_{0}}\right)}^{-1}T_{b}^{i_{0}}=\left[\begin{array}{cc} C_{i_{0}}^{b^{\prime }} & -C_{i_{0}}^{b^{\prime }}{\hat{v}}_{f}^{i_{0}}\\ 0_{1\times 3} & 1 \end{array}\right]\left[\begin{array}{cc} C_{b}^{i_{0}} & v_{f}^{i_{0}}\\ 0_{1\times 3} & 1 \end{array}\right]\\ \; & =\left[\begin{array}{cc} C_{b}^{b^{\prime }} & C_{i_{0}}^{b^{\prime }}\left(v_{f}^{i_{0}}-{\hat{v}}_{f}^{i_{0}}\right)\\ 0_{1\times 3} & 1 \end{array}\right]≜\left[\begin{array}{cc} C_{b}^{b^{\prime }} & {\left(\delta v_{f}\right)}_{l}\\ 0_{1\times 3} & 1 \end{array}\right] \end{array}$$

where $T_{b}^{i_{0}}$ is the description of the true motion of the *b*-frame relative to the $i_{0}$-frame on the SE(3) group, ${\hat{T}}_{b}^{i_{0}}$ is its computational solution, the $b^{\prime }$-frame is the computational body coordinate system, $C_{b}^{b^{\prime }}$ is the left-invariant attitude error matrix of the *b*-frame, and ${\left(\delta v_{f}\right)}_{l}$ is the left-invariant specific force velocity error.

Applying a differential operation to the above equation, we obtain

(13) $${\dot{\delta }}_{l}^{i_{0}b}=\left[\begin{array}{cc} {\dot{C}}_{b}^{b^{\prime }} & {\left(\delta {\dot{v}}_{f}\right)}_{l}\\ 0_{1\times 3} & 0 \end{array}\right]$$

(14) $${\dot{C}}_{b}^{b^{\prime }}=C_{b}^{b^{\prime }}\left(\omega_{b^{\prime }b}^{b}\times \right)$$

(15) $$\omega_{b^{\prime }b}^{b}={\hat{\omega }}_{i_{0}b}^{b}-\delta \omega_{i_{0}b}^{b}-C_{b^{\prime }}^{b}{\hat{\omega }}_{i_{0}b}^{b}=\left(I-C_{b^{\prime }}^{b}\right){\hat{\omega }}_{i_{0}b}^{b}-\delta \omega_{i_{0}b}^{b}$$

where $\omega_{b^{\prime }b}^{b}$ is the projection of the angular velocity of the *b*-frame relative to the $b^{\prime }$-frame in the *b*-frame, ${\hat{\omega }}_{i_{0}b}^{b}$ is the measured value provided by the gyroscope, and $\delta \omega_{i_{0}b}^{b}$ is the gyroscope’s error.

(16) $$\begin{array}{cc} {\left(\delta {\dot{v}}_{f}\right)}_{l} & =\frac{d\left[C_{i_{0}}^{b^{\prime }}\left(v_{f}^{i_{0}}-{\hat{v}}_{f}^{i_{0}}\right)\right]}{dt}=\frac{d\left(C_{b}^{b^{\prime }}v_{f}^{b}-{\hat{v}}_{f}^{b}\right)}{dt}\\ \; & =C_{b}^{b^{\prime }}\left(\omega_{b^{\prime }b}^{b}\times \right)v_{f}^{b}+C_{b}^{b^{\prime }}f^{b}-{\hat{f}}^{b}\\ \; & =C_{b}^{b^{\prime }}\left(\omega_{b^{\prime }b}^{b}\times \right)C_{b^{\prime }}^{b}\left[{\hat{v}}_{f}^{b}+{\left(\delta v_{f}\right)}_{l}\right]+C_{b}^{b^{\prime }}\left({\hat{f}}^{b}-\delta f^{b}\right)-{\hat{f}}^{b}\\ \; & =C_{b}^{b^{\prime }}\left(\omega_{b^{\prime }b}^{b}\times \right)C_{b^{\prime }}^{b}\left[{\hat{v}}_{f}^{b}+{\left(\delta v_{f}\right)}_{l}\right]-\left(I-C_{b}^{b^{\prime }}\right){\hat{f}}^{b}-C_{b}^{b^{\prime }}\delta f^{b} \end{array}$$

where ${\hat{v}}_{f}^{b}$ is the projection of the computational specific force velocity in the $b^{\prime }$-frame (For simplicity, ${\hat{v}}_{f}^{b^{\prime }}≜{\hat{v}}_{f}^{b}$.), ${\hat{f}}^{b}$ is the measured value provided by the accelerometer, $\delta f^{b}$ is the accelerometer’s error, and $f^{b}$ is the true specific force.

Therefore, the kinematic equation of $\delta_{l}^{i_{0}b}$ is

(17) $${\dot{\delta }}_{l}^{i_{0}b}=\left[\begin{array}{cc} C_{b}^{b^{\prime }} & {\left(\delta v_{f}\right)}_{l}\\ 0_{1\times 3} & 1 \end{array}\right]\left[\begin{array}{cc} \left(\omega_{b^{\prime }b}^{b}\times \right) & {\left(\delta a_{f}\right)}_{l}\\ 0_{1\times 3} & 0 \end{array}\right]≜\delta_{l}^{i_{0}b}\Xi_{l}^{i_{0}b}$$

where $\Xi_{l}^{i_{0}b}$ is the related Lie algebra to $\delta_{l}^{i_{0}b}$, and ${\left(\delta a_{f}\right)}_{l}$ is determined by (18).

(18) $$\begin{array}{cc} {\left(\delta a_{f}\right)}_{l} & =C_{b^{\prime }}^{b}{\left(\delta {\dot{v}}_{f}\right)}_{l}\\ \; & =\left(\omega_{b^{\prime }b}^{b}\times \right)C_{b^{\prime }}^{b}\left[{\hat{v}}_{f}^{b}+{\left(\delta v_{f}\right)}_{l}\right]+\left(I-C_{b^{\prime }}^{b}\right){\hat{f}}^{b}-\delta f^{b} \end{array}$$

In the same way, the left-invariant error of $T_{n}^{i_{0}}$ is

(19) $$\begin{array}{cc} \delta_{l}^{i_{0}n} & ={\left({\hat{T}}_{n}^{i_{0}}\right)}^{-1}T_{n}^{i_{0}}=\left[\begin{array}{cc} C_{i_{0}}^{n^{\prime }} & -C_{i_{0}}^{n^{\prime }}{\hat{v}}_{g}^{i_{0}}\\ 0_{1\times 3} & 1 \end{array}\right]\left[\begin{array}{cc} C_{n}^{i_{0}} & v_{g}^{i_{0}}\\ 0_{1\times 3} & 1 \end{array}\right]\\ \; & =\left[\begin{array}{cc} C_{n}^{n^{\prime }} & C_{i_{0}}^{n^{\prime }}\left(v_{g}^{i_{0}}-{\hat{v}}_{g}^{i_{0}}\right)\\ 0_{1\times 3} & 1 \end{array}\right]≜\left[\begin{array}{cc} C_{n}^{n^{\prime }} & {\left(\delta v_{g}\right)}_{l}\\ 0_{1\times 3} & 1 \end{array}\right] \end{array}$$

where $T_{n}^{i_{0}}$ is the description of the true motion of the *n*-frame relative to the $i_{0}$-frame on the SE(3) group, ${\hat{T}}_{n}^{i_{0}}$ is its computational solution, the $n^{\prime }$-frame is the computational geographic coordinate system, $C_{n}^{n^{\prime }}$ is the left-invariant attitude error matrix of the *n*-frame, and ${\left(\delta v_{g}\right)}_{l}$ is the left-invariant gravity velocity error.

Thus, one can get the following differential equations.

(20) $$\begin{array}{cc} {\dot{\delta }}_{l}^{i_{0}n} & =\left[\begin{array}{cc} {\dot{C}}_{n}^{n^{\prime }} & {\left(\delta {\dot{v}}_{g}\right)}_{l}\\ 0_{1\times 3} & 0 \end{array}\right] \end{array}$$

(21) $$\begin{array}{cc} {\dot{C}}_{n}^{n^{\prime }} & =C_{n}^{n^{\prime }}\left(\omega_{n^{\prime }n}^{n}\times \right) \end{array}$$

(22) $$\begin{array}{cc} \omega_{n^{\prime }n}^{n} & =\left(I-C_{n^{\prime }}^{n}\right){\hat{\omega }}_{i_{0}n}^{n}-\delta \omega_{i_{0}n}^{n} \end{array}$$

(23) $$\begin{array}{cc} {\left(\delta {\dot{v}}_{g}\right)}_{l} & =\frac{d\left[C_{i_{0}}^{n^{\prime }}\left(v_{g}^{i_{0}}-{\hat{v}}_{g}^{i_{0}}\right)\right]}{dt}=\frac{d\left(C_{n}^{n^{\prime }}v_{g}^{n}-{\hat{v}}_{g}^{n}\right)}{dt}\\ \; & =C_{n}^{n^{\prime }}\left(\omega_{n^{\prime }n}^{n}\times \right)C_{n^{\prime }}^{n}\left[{\hat{v}}_{g}^{n}+{\left(\delta v_{g}\right)}_{l}\right]-\left(I-C_{n}^{n^{\prime }}\right){\hat{g}}^{n}-C_{n}^{n^{\prime }}\delta g^{n} \end{array}$$

where $\omega_{n^{\prime }n}^{n}$ is the projection of the angular velocity of the *n*-frame relative to the $n^{\prime }$-frame in the *n*-frame. ${\hat{\omega }}_{i_{0}n}^{n}$ is the computational projection of the angular velocity of the $n^{\prime }$-frame with respect to the $i_{0}$-frame in the $n^{\prime }$-frame, which is calculated by the computational velocity and position, and $\delta \omega_{i_{0}n}^{n}$ is its error. ${\hat{v}}_{g}^{n}$ is the computational gravity velocity in the $n^{\prime }$-frame (For simplicity, ${\hat{v}}_{g}^{n^{\prime }}≜{\hat{v}}_{g}^{n}$.). ${\hat{g}}^{n}$ is the computational gravity vector calculated by the computational position and normal gravity model, and $\delta g^{n}$ is the gravity disturbance vector.

Consequently, the kinematic equation of $\delta_{l}^{i_{0}n}$ is

(24) $${\dot{\delta }}_{l}^{i_{0}n}=\left[\begin{array}{cc} C_{n}^{n^{\prime }} & {\left(\delta v_{g}\right)}_{l}\\ 0_{1\times 3} & 1 \end{array}\right]\left[\begin{array}{cc} \left(\omega_{n^{\prime }n}^{n}\times \right) & {\left(\delta a_{g}\right)}_{l}\\ 0_{1\times 3} & 0 \end{array}\right]≜\delta_{l}^{i_{0}n}\Xi_{l}^{i_{0}n}$$

where $\Xi_{l}^{i_{0}n}$ is the related Lie algebra to $\delta_{l}^{i_{0}n}$ and ${\left(\delta a_{g}\right)}_{l}$ is determined by (25).

(25) $$\begin{array}{cc} {\left(\delta a_{g}\right)}_{l} & =C_{n^{\prime }}^{n}{\left(\delta {\dot{v}}_{g}\right)}_{l}=\left(\omega_{n^{\prime }n}^{n}\times \right)C_{n^{\prime }}^{n}\left[{\hat{v}}_{g}^{n}+{\left(\delta v_{g}\right)}_{l}\right]+\left(I-C_{n^{\prime }}^{n}\right){\hat{g}}^{n}-\delta g^{n} \end{array}$$

### 3.2. Right-Invariant Error Definition and Kinematics

By virtue of the determinacy of the $i_{0}$-frame, the attitude error completely exists in the orientation of the *b*-frame, so the Lie group right-invariant error of $T_{b}^{i_{0}}$ is defined as

(26) $$\begin{array}{cc} \delta_{r}^{i_{0}b} & =T_{i_{0}}^{b}{\left({\hat{T}}_{i_{0}}^{b}\right)}^{-1}=\left[\begin{array}{cc} C_{i_{0}}^{b} & v_{f}^{b}\\ 0_{1\times 3} & 1 \end{array}\right]\left[\begin{array}{cc} C_{b^{\prime }}^{i_{0}} & -C_{b^{\prime }}^{i_{0}}{\hat{v}}_{f}^{b}\\ 0_{1\times 3} & 1 \end{array}\right]\\ \; & =\left[\begin{array}{cc} C_{b^{\prime }}^{b} & v_{f}^{b}-C_{b^{\prime }}^{b}{\hat{v}}_{f}^{b}\\ 0_{1\times 3} & 1 \end{array}\right]≜\left[\begin{array}{cc} C_{b^{\prime }}^{b} & {\left(\delta v_{f}\right)}_{r}\\ 0_{1\times 3} & 1 \end{array}\right] \end{array}$$

where $C_{b^{\prime }}^{b}$ is the right-invariant attitude error matrix of the *b*-frame and ${\left(\delta v_{f}\right)}_{r}$ is the right-invariant specific force velocity error.

Taking the derivative of (26), one can get

(27) $${\dot{\delta }}_{r}^{i_{0}b}=\left[\begin{array}{cc} {\dot{C}}_{b^{\prime }}^{b} & {\left(\delta {\dot{v}}_{f}\right)}_{r}\\ 0_{1\times 3} & 0 \end{array}\right]$$

(28) $${\dot{C}}_{b^{\prime }}^{b}=C_{b^{\prime }}^{b}\left(\omega_{bb^{\prime }}^{b^{\prime }}\times \right)$$

(29) $$\begin{array}{cc} \omega_{bb^{\prime }}^{b^{\prime }} & ={\hat{\omega }}_{i_{0}b}^{b}-C_{b}^{b^{\prime }}\omega_{i_{0}b}^{b}={\hat{\omega }}_{i_{0}b}^{b}-C_{b}^{b^{\prime }}\left({\hat{\omega }}_{i_{0}b}^{b}-\delta \omega_{i_{0}b}^{b}\right)\\ \; & =\left(I-C_{b}^{b^{\prime }}\right){\hat{\omega }}_{i_{0}b}^{b}+C_{b}^{b^{\prime }}\delta \omega_{i_{0}b}^{b} \end{array}$$

where $\omega_{bb^{\prime }}^{b^{\prime }}$ is the projection of the angular velocity of the $b^{\prime }$-frame relative to the *b*-frame in the $b^{\prime }$-frame and $\omega_{i_{0}b}^{b}$ is the true projection of the angular velocity of the *b*-frame relative to the $i_{0}$-frame in the *b*-frame.

(30) $$\begin{array}{cc} {\left(\delta {\dot{v}}_{f}\right)}_{r} & =\frac{d\left(v_{f}^{b}-C_{b^{\prime }}^{b}{\hat{v}}_{f}^{b}\right)}{dt}\\ \; & =f^{b}-C_{b^{\prime }}^{b}\left(\omega_{bb^{\prime }}^{b^{\prime }}\times \right){\hat{v}}_{f}^{b}-C_{b^{\prime }}^{b}{\hat{f}}^{b}\\ \; & ={\hat{f}}^{b}-\delta f^{b}-C_{b^{\prime }}^{b}\left(\omega_{bb^{\prime }}^{b^{\prime }}\times \right){\hat{v}}_{f}^{b}-C_{b^{\prime }}^{b}{\hat{f}}^{b}\\ \; & =\left(I-C_{b^{\prime }}^{b}\right){\hat{f}}^{b}-C_{b^{\prime }}^{b}\left(\omega_{bb^{\prime }}^{b^{\prime }}\times \right){\hat{v}}_{f}^{b}-\delta f^{b} \end{array}$$

Therefore, the kinematic equation of $\delta_{r}^{i_{0}b}$ is given by

(31) $${\dot{\delta }}_{r}^{i_{0}b}=\left[\begin{array}{cc} C_{b^{\prime }}^{b} & {\left(\delta v_{f}\right)}_{r}\\ 0_{1\times 3} & 1 \end{array}\right]\left[\begin{array}{cc} \left(\omega_{bb^{\prime }}^{b^{\prime }}\times \right) & {\left(\delta a_{f}\right)}_{r}\\ 0_{1\times 3} & 0 \end{array}\right]≜\delta_{r}^{i_{0}b}\Xi_{r}^{i_{0}b}$$

where $\Xi_{r}^{i_{0}b}$ is the related Lie algebra to $\delta_{r}^{i_{0}b}$ and ${\left(\delta a_{f}\right)}_{r}$ is determined by (32).

(32) $$\begin{array}{cc} {\left(\delta a_{f}\right)}_{r} & =C_{b}^{b^{\prime }}{\left(\delta {\dot{v}}_{f}\right)}_{r}\\ \; & =-\left(I-C_{b}^{b^{\prime }}\right){\hat{f}}^{b}-\left(\omega_{bb^{\prime }}^{b^{\prime }}\times \right){\hat{v}}_{f}^{b}-C_{b}^{b^{\prime }}\delta f^{b} \end{array}$$

Similarly, the right-invariant error of $T_{i_{0}}^{n}$ is

(33) $$\begin{array}{cc} \delta_{r}^{i_{0}n} & =T_{i_{0}}^{n}{\left({\hat{T}}_{i_{0}}^{n}\right)}^{-1}=\left[\begin{array}{cc} C_{i_{0}}^{n} & v_{g}^{n}\\ 0_{1\times 3} & 1 \end{array}\right]\left[\begin{array}{cc} C_{n^{\prime }}^{i_{0}} & -C_{n^{\prime }}^{i_{0}}{\hat{v}}_{g}^{n}\\ 0_{1\times 3} & 1 \end{array}\right]\\ \; & =\left[\begin{array}{cc} C_{n^{\prime }}^{n} & v_{g}^{n}-C_{n^{\prime }}^{n}{\hat{v}}_{g}^{n}\\ 0_{1\times 3} & 1 \end{array}\right]≜\left[\begin{array}{cc} C_{n^{\prime }}^{n} & {\left(\delta v_{g}\right)}_{r}\\ 0_{1\times 3} & 1 \end{array}\right] \end{array}$$

where $C_{n^{\prime }}^{n}$ is the right-invariant attitude error matrix of the *n*-frame and ${\left(\delta v_{g}\right)}_{r}$ is the right-invariant gravity velocity error.

The kinematics of $\delta_{r}^{i_{0}n}$ are demonstrated by the following equations.

(34) $${\dot{\delta }}_{r}^{i_{0}n}=\left[\begin{array}{cc} {\dot{C}}_{n^{\prime }}^{n} & {\left(\delta {\dot{v}}_{g}\right)}_{r}\\ 0_{1\times 3} & 0 \end{array}\right]$$

(35) $${\dot{C}}_{n^{\prime }}^{n}=C_{n^{\prime }}^{n}\left(\omega_{nn^{\prime }}^{n^{\prime }}\times \right)$$

(36) $$\omega_{nn^{\prime }}^{n^{\prime }}=\left(I-C_{n}^{n^{\prime }}\right){\hat{\omega }}_{i_{0}n}^{n}+C_{n}^{n^{\prime }}\delta \omega_{i_{0}n}^{n}$$

where $\omega_{nn^{\prime }}^{n^{\prime }}$ is the projection of the angular velocity of the $n^{\prime }$-frame relative to the *n*-frame in the $n^{\prime }$-frame.

(37) $$\begin{array}{cc} {\left(\delta {\dot{v}}_{g}\right)}_{r} & =\frac{d\left(v_{g}^{n}-C_{n^{\prime }}^{n}{\hat{v}}_{g}^{n}\right)}{dt}\\ \; & =\left(I-C_{n^{\prime }}^{n}\right){\hat{g}}^{n}-C_{n^{\prime }}^{n}\left(\omega_{nn^{\prime }}^{n^{\prime }}\times \right){\hat{v}}_{g}^{n}-\delta g^{n} \end{array}$$

Therefore, the kinematics of $\delta_{r}^{i_{0}n}$ is

(38) $${\dot{\delta }}_{r}^{i_{0}n}=\left[\begin{array}{cc} C_{n^{\prime }}^{n} & {\left(\delta v_{g}\right)}_{r}\\ 0_{1\times 3} & 1 \end{array}\right]\left[\begin{array}{cc} \left(\omega_{nn^{\prime }}^{n^{\prime }}\times \right) & {\left(\delta a_{g}\right)}_{r}\\ 0_{1\times 3} & 0 \end{array}\right]≜\delta_{r}^{i_{0}n}\Xi_{r}^{i_{0}n}$$

where $\Xi_{r}^{i_{0}n}$ is the related Lie algebra to $\delta_{r}^{i_{0}n}$ and ${\left(\delta a_{g}\right)}_{r}$ is calculated by (39).

(39) $$\begin{array}{cc} {\left(\delta a_{g}\right)}_{r} & =C_{n}^{n^{\prime }}{\left(\delta {\dot{v}}_{g}\right)}_{r}\\ \; & =-\left(I-C_{n}^{n^{\prime }}\right){\hat{g}}^{n}-\left(\omega_{nn^{\prime }}^{n^{\prime }}\times \right){\hat{v}}_{g}^{n}-C_{n}^{n^{\prime }}\delta g^{n} \end{array}$$

**Remark** **2.** *By constructing and solving the kinematic equations of the n-frame and the b-frame with respect to the deterministic $i_{0}$-frame and their associated invariant errors on the SE(3) group, navigation parameter errors and sensor errors are separated and decoupled. Specifically, the errors of the b-frame are entirely contingent on the SINS errors, as shown in (15), (16), (29) and (30), while the errors of the n-frame depend on the errors of the computational velocity and position, as shown in (22), (23), (36) and (37). Compared with traditional methods that are established on the assumption that the computational $n^{\prime }$-frame and n-frame coincide, or that the computational $b^{\prime }$-frame and b-frame coincide, the $i_{0}$-frame as a reliable benchmark, provides a more reasonable and specific way to define the dynamic and its errors of the n-frame and the b-frame, and clarifies the evolution trajectories of the errors on the differentiable manifold and elucidates the factors affecting the dynamic more explicitly.*

**Remark** **3.** *Because this study is based on the premise that the $i_{0}$-frame is accurate and stationary, there is no essential difference between the left- and right-invariant attitude error matrices (related by transposition), but there is a slight difference in the representation of the velocity errors. The left-invariant velocity errors are projected in the computational $n^{\prime }$-frame and the computational $b^{\prime }$-frame, whereas the right-invariant velocity errors are located in the n-frame and the b-frame. However, both models take the errors brought by the inconsistency of the reference coordinate system into account, and fundamentally equivalent.*

### 3.3. The Application of the Error Kinematics in the Filtering

According to the error kinematics derived above, the following state model can be built.

(40) $$\begin{array}{cc} \; & x=\left(\delta_{j}^{i_{0}b},\delta_{j}^{i_{0}n},\delta v_{corr}^{n},\delta p,b_{g},b_{a}\right)\in \mathrm{SE}\left(3\right)\times \mathrm{SE}\left(3\right)\times R^{12},\;j=\begin{array}{ccc} l & \mathrm{or} & r \end{array} \end{array}$$

where $\delta v_{corr}^{n}$ is the Coriolis velocity error, δp is the position error, and $b_{g}$ and $b_{a}$ are the constant drifts of the gyroscope and the constant biases of the accelerometer, respectively.

In order to apply the KF or some nonlinear variants of it to estimate the above state variable, considering the correspondence between attitude matrices and Euler angles, the 24-dimensional state vectors based on the Lie group left- and right-invariant error models presented below are introduced.

(41) $$\begin{array}{cc} X_{l}= & \left[{\left(\varphi_{b}^{b^{\prime }}\right)}^{T},{\left(\varphi_{n}^{n^{\prime }}\right)}^{T},{\left(\delta v_{f}\right)}_{l}^{T},{\left(\delta v_{g}\right)}_{l}^{T},\right.{\left.{\left(\delta v_{corr}^{n}\right)}^{T},{\left(\delta p\right)}^{T},b_{g}^{T},b_{a}^{T}\right]}^{T}\in R^{24} \end{array}$$

(42) $$\begin{array}{cc} X_{r}= & \left[{\left(\varphi_{b^{\prime }}^{b}\right)}^{T},{\left(\varphi_{n^{\prime }}^{n}\right)}^{T},{\left(\delta v_{f}\right)}_{r}^{T},{\left(\delta v_{g}\right)}_{r}^{T},\right.{\left.{\left(\delta v_{corr}^{n}\right)}^{T},{\left(\delta p\right)}^{T},b_{g}^{T},b_{a}^{T}\right]}^{T}\in R^{24} \end{array}$$

where $\varphi_{b}^{b^{\prime }}$, $\varphi_{n}^{n^{\prime }}$, $\varphi_{b^{\prime }}^{b}$, and $\varphi_{n^{\prime }}^{n}$ are the Euler error angles corresponding to the attitude error matrices $C_{b}^{b^{\prime }}$, $C_{n}^{n^{\prime }}$, $C_{b^{\prime }}^{b}$, and $C_{n^{\prime }}^{n}$, respectively.

The kinematic equation of the Coriolis velocity error can be obtained by differentiating (7), which is

(43) $$\delta {\dot{v}}_{corr}^{n}=-\left({\hat{\omega }}_{i_{0}e}^{n}+{\hat{\omega }}_{i_{0}n}^{n}\right)\times \delta v^{n}-\left(\delta \omega_{i_{0}e}^{n}+\delta \omega_{i_{0}n}^{n}\right)\times {\hat{v}}^{n}$$

where ${\hat{\omega }}_{i_{0}e}^{n}$ is the computational Earth’s angular velocity in the $n^{\prime }$-frame according to the computational position and $\delta \omega_{i_{0}e}^{n}$ is its error. $\delta v^{n}$ is the velocity error in the *n*-frame, and it can be calculated by the following equations.

(44) $$\begin{array}{cc} \delta v^{n} & ≜{\hat{v}}^{n}-v^{n}=\left(C_{b^{\prime }}^{n^{\prime }}{\hat{v}}_{f}^{b}-C_{b}^{n}v_{f}^{b}\right)+\left({\hat{v}}_{g}^{n}-v_{g}^{n}\right)+\left({\hat{v}}_{corr}^{n}-v_{corr}^{n}\right) \end{array}$$

(45) $$\begin{array}{cc} \delta v_{l}^{n} & =\left\{C_{b^{\prime }}^{n^{\prime }}{\hat{v}}_{f}^{b}-C_{b}^{n}\left[C_{b^{\prime }}^{b}\left({\hat{v}}_{f}^{b}+{\left(\delta v_{f}\right)}_{l}\right)\right]\right\}+\left\{{\hat{v}}_{g}^{n}-C_{n^{\prime }}^{n}\left[{\hat{v}}_{g}^{n}+{\left(\delta v_{g}\right)}_{l}\right]\right\}+\delta v_{corr}^{n}\\ \; & =\left(I-C_{n^{\prime }}^{n}\right)C_{b^{\prime }}^{n^{\prime }}{\hat{v}}_{f}^{b}-C_{n^{\prime }}^{n}C_{b^{\prime }}^{n^{\prime }}{\left(\delta v_{f}\right)}_{l}+\left(I-C_{n^{\prime }}^{n}\right){\hat{v}}_{g}^{n}-C_{n^{\prime }}^{n}{\left(\delta v_{g}\right)}_{l}+\delta v_{corr}^{n} \end{array}$$

(46) $$\begin{array}{cc} \delta v_{r}^{n} & =\left\{C_{b^{\prime }}^{n^{\prime }}{\hat{v}}_{f}^{b}-C_{b}^{n}\left[C_{b^{\prime }}^{b}{\hat{v}}_{f}^{b}+{\left(\delta v_{f}\right)}_{r}\right]\right\}+\left\{{\hat{v}}_{g}^{n}-\left[C_{n^{\prime }}^{n}{\hat{v}}_{g}^{n}+{\left(\delta v_{g}\right)}_{r}\right]\right\}+\delta v_{corr}^{n}\\ \; & =\left(I-C_{n^{\prime }}^{n}\right)C_{b^{\prime }}^{n^{\prime }}{\hat{v}}_{f}^{b}-C_{n^{\prime }}^{n}C_{b^{\prime }}^{n^{\prime }}C_{b^{\prime }}^{b}{\left(\delta v_{f}\right)}_{r}+\left(I-C_{n^{\prime }}^{n}\right){\hat{v}}_{g}^{n}-{\left(\delta v_{g}\right)}_{r}+\delta v_{corr}^{n} \end{array}$$

where $\delta v_{l}^{n}$ is the defined velocity error under the left-invariant error model, $\delta v_{r}^{n}$ is the one under the right-invariant error model, and $C_{b^{\prime }}^{n^{\prime }}$ is the computational attitude matrix between the $n^{\prime }$-frame and the $b^{\prime }$-frame.

Hence, the following state transition model can be acquired.

(47) $${\dot{X}}_{j}=f_{j}\left(X_{j}\right)+G_{j}w,j=\begin{array}{ccc} l & \mathrm{or} & r \end{array}$$

where $f_{j}$ is the transition function derived in the last section, $G_{j}$ is the noise input matrix, which is given by (48), and w is the noise vector mainly including the gyroscope random noise $w_{g}={\left[\begin{array}{ccc} w_{gx} & w_{gy} & w_{gz} \end{array}\right]}^{T}$ and the accelerometer random noise $w_{a}={\left[\begin{array}{ccc} w_{ax} & w_{ay} & w_{az} \end{array}\right]}^{T}$ (*x*, *y*, and *z* represent the three axes of the inertial sensors), both of which are normally modeled as a Gaussian white noise.

(48) $$G_{l}=\left[\begin{array}{c} \begin{array}{cc} -I_{3} & 0_{3\times 3} \end{array}\\ 0_{3\times 6}\\ \begin{array}{cc} 0_{3\times 3} & -C_{b}^{b^{\prime }} \end{array}\\ 0_{15\times 6} \end{array}\right],G_{r}=\left[\begin{array}{c} \begin{array}{cc} C_{b}^{b^{\prime }} & 0_{3\times 3} \end{array}\\ 0_{3\times 6}\\ \begin{array}{cc} 0_{3\times 3} & -I_{3} \end{array}\\ 0_{15\times 6} \end{array}\right]$$

The observation vector of velocity is given as

(49) $$Z_{v}={\hat{v}}^{n}-v_{obs}^{n}$$

where $v_{obs}^{n}$ is the velocity measurements provided by other navigation systems.

Correspondingly, the feedback model is

(50) $$v^{n}={\hat{v}}^{n}-\delta v_{j}^{n},j=\begin{array}{ccc} l & \mathrm{or} & r \end{array}$$

## 4. Experiments

In this section, the data from a turntable experiment and a car-mounted field experiment are processed to verify the effectiveness of the proposed models for attitude determination. The results are compared with those calculated by the traditional SO(3) group model and the conventional SE(3) group model. Zhu et al. pointed out the equivalence between the left- and right-invariant error models [14], so only the right-invariant error model on the SE(3) group are compared. In the experiment, the proposed left- and right-invariant error models on the SE(3) group in the ECI frame and the two models mentioned before are named as ECI-SE(3)-L model, ECI-SE(3)-R model, SO(3) model, and SE(3) model, respectively.

### 4.1. Turntable Experiment

The validity of the proposed models in improving the attitude accuracy was first confirmed through a turntable experiment. A three-axis high-precision turntable was used, initially orientated in the East–North–Up direction, and following a short period of rest, the inner, middle, and outer frames of the turntable swayed with the amplitudes of $3^{\circ }$, $3^{\circ }$, and $6^{\circ }$, and the frequency of 0.1 Hz, respectively. After approximately 1900 s of oscillation, the turntable returned to a stationary state. A navigation-level SINS was installed at the center of the turntable, consisting of a high-precision triaxial laser gyroscope with a drift rate better than 0.005°/h, random noise of about 0.001°/$\sqrt{h}$, and a triaxial accelerometer with a bias of less than 50 μg. In addition, a GNSS was employed to provide velocity and position information, with a velocity accuracy of about 0.1 m/s and a position accuracy of about 10 m. The sampling rates of the SINS and the GNSS were 200 Hz and 1 Hz, respectively. The main parameters of the sensors are listed in Table 1.

We tested and analyzed three cases with the initial misalignment angles set to [$0^{\circ }$, $0^{\circ }$, $0^{\circ }$], [$2^{\circ }$, $2^{\circ }$, $10^{\circ }$], and [$10^{\circ }$, $10^{\circ }$, $50^{\circ }$] (corresponding to the pitch, roll, and yaw directions, respectively).

**Figure 1:**
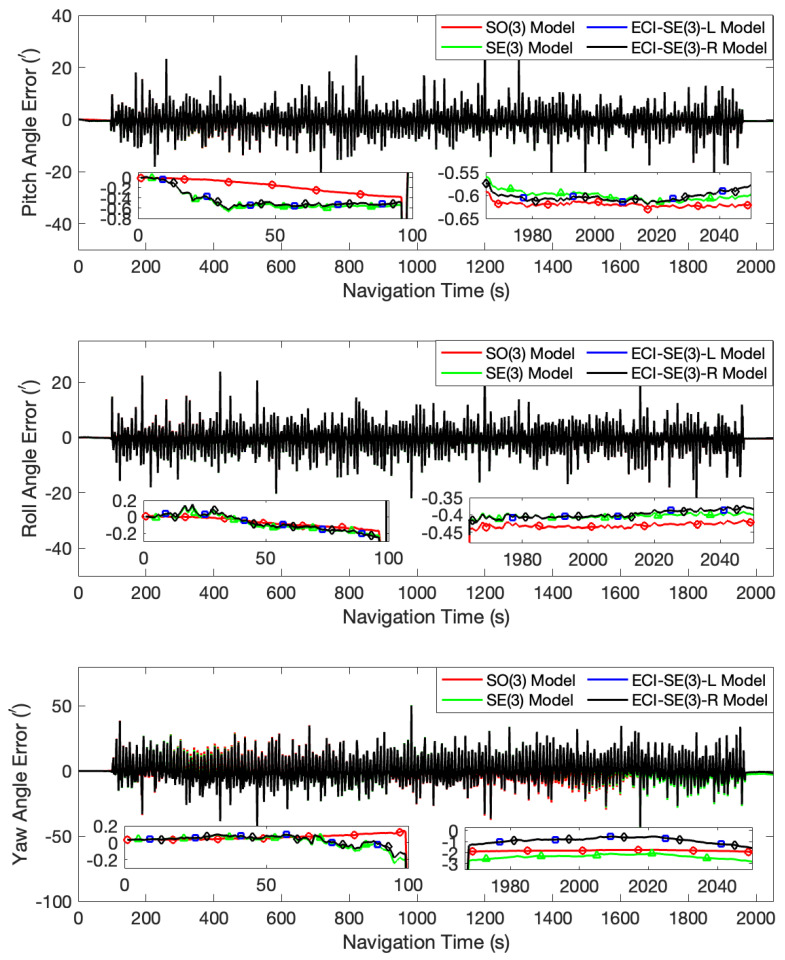
The attitude angle error curves at the initial misalignment angles of [$0^{\circ }$, $0^{\circ }$, $0^{\circ }$].

In the first case, with initial misalignment angles of [$0^{\circ }$, $0^{\circ }$, $0^{\circ }$], all the four models quickly converged and accurately tracked the attitude changes of the turntable. The error curves of this test are shown in Figure 1. After the turntable returned to a stationary state, the pitch angle errors calculated by the four models were all between $0.56^{\prime }$ and $0.65^{\prime }$, and the roll angle errors were all between $0.37^{\prime }$ and $0.46^{\prime }$. Regarding yaw angle errors, the proposed left- and right-invariant error models had a slight advantage, with an error of approximately $1.25^{\prime }$, compared to errors of $1.89^{\prime }$ and $2.66^{\prime }$ for the SO(3) and SE(3) models, respectively. In addition, in the early stage of filtering, the error curves for the SE(3), the ECI-SE(3)-L, and the ECI-SE(3)-R models exhibited greater fluctuations and larger deviations from the true value compared to the SO(3) model, which is due to the overestimation of common frame errors in the error-free stationary state. In this specific instance, the initial attitude is absolutely accurate. However, under the Lie group invariant error definition, the nonexistent attitude error matrix is considered and coupled into the invariant velocity error. During the filtering process, error allocation is performed according to the Kalman filtering principle, which assigns unexpected errors to the attitude and consequently deteriorates the estimation effect instead. On the contrary, for large misalignment angles, the Lie group invariant error models effectively resolve the common frame error, allowing for the rapid correction of the misalignment angles. This effectiveness is also demonstrated in subsequent experimental results. During the turntable oscillation, the attitude angle error produced periodic error terms at the same frequency as the oscillation, which may be generated by the arm lever.

**Figure 2:**
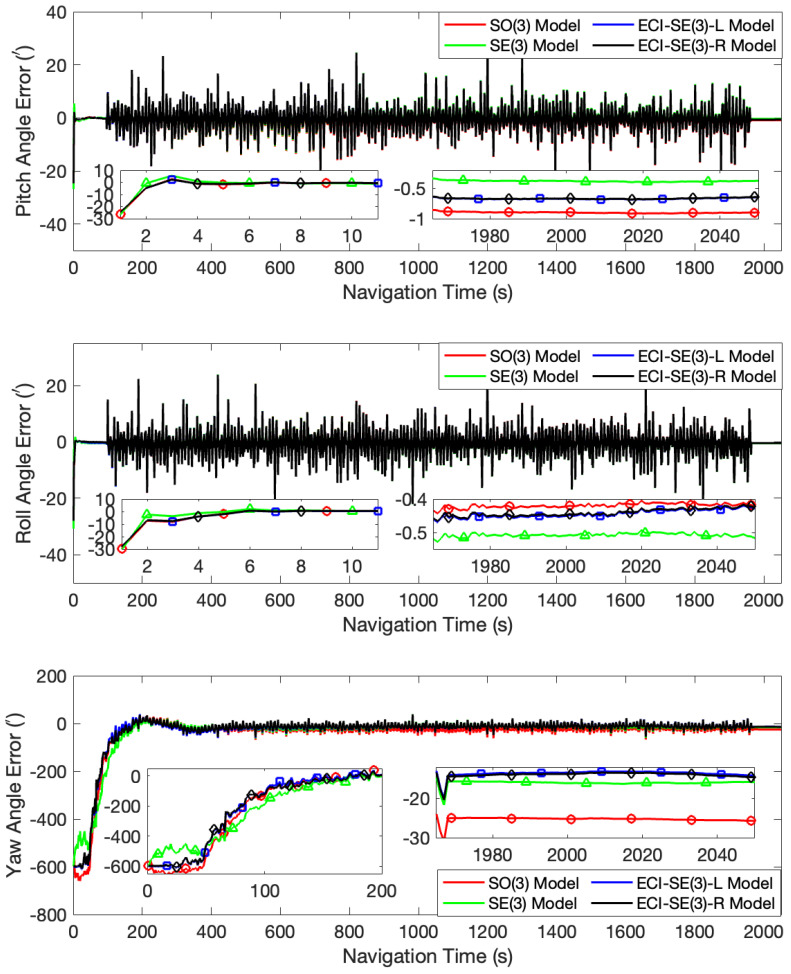
The attitude angle error curves at the initial misalignment angles of [$2^{\circ }$, $2^{\circ }$, $10^{\circ }$].

When the initial misalignment angles were set to [$2^{\circ }$, $2^{\circ }$, $10^{\circ }$], all four models achieved satisfactory accuracy in estimating the two horizontal attitude angles. The SO(3) model, SE(3) model, ECI-SE(3)-L model, and ECI-SE(3)-R model obtained pitch angle errors of $0.86^{\prime }$, $0.34^{\prime }$, $0.64^{\prime }$, and $0.64^{\prime }$, respectively, and roll angle errors of $0.44^{\prime }$, $0.52^{\prime }$, $0.47^{\prime }$, and $0.46^{\prime }$, respectively, after the turntable completed its oscillation. However, there was a significant deviation in estimating the yaw angle for the SO(3) model, with an error of $25.16^{\prime }$. Additionally, the proposed models presented more pronounced advantages compared to the SE(3) model. Specifically, the yaw angle errors of the SE(3) model, the ECI-SE(3)-L model, and the ECI-SE(3)-R model were $15.79^{\prime }$, $14.07^{\prime }$, and $14.47^{\prime }$, respectively. The error curves for these tests are presented in Figure 2.

**Figure 3:**
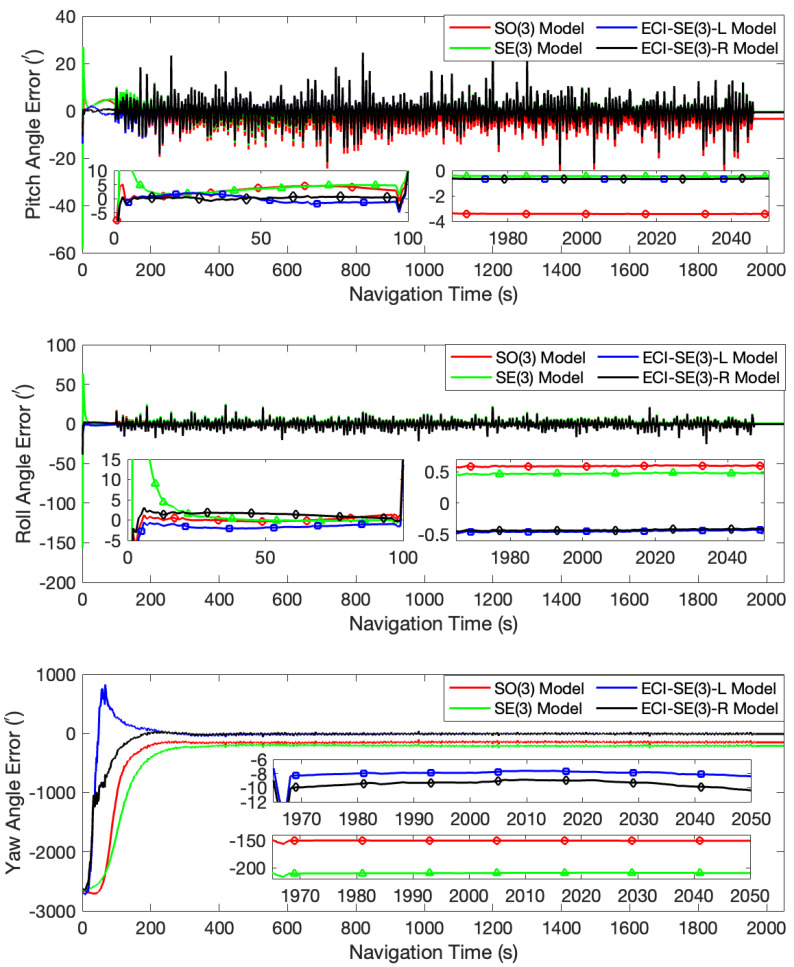
The attitude angle error curves at the initial misalignment angles of [$10^{\circ }$, $10^{\circ }$, $50^{\circ }$].

**Figure 4:**
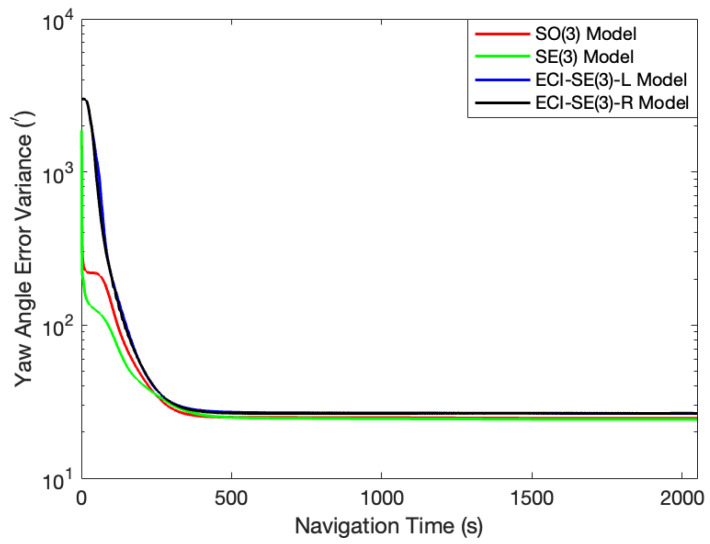
The variance of the yaw angle error at the initial misalignment angles of [$10^{\circ }$, $10^{\circ }$, $50^{\circ }$].

**Figure 5:**
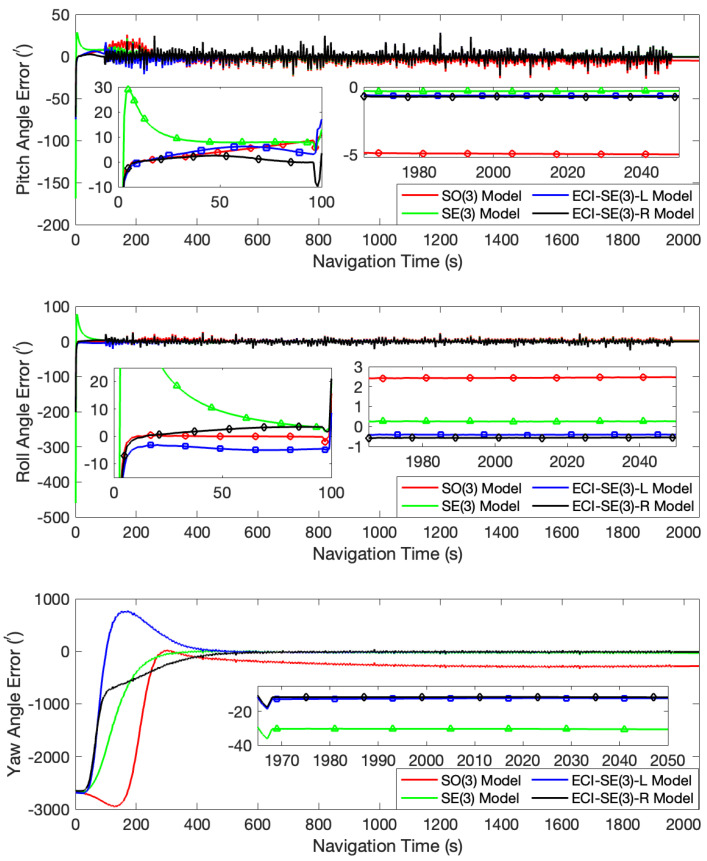
The attitude angle error curves at the initial misalignment angles of $10^{\circ }$, $10^{\circ }$, $50^{\circ }$ after the observation variance adjustment.

Under the condition of an initial misalignment angle of [$10^{\circ }$, $10^{\circ }$, $50^{\circ }$], different outcomes were observed. While the four models maintained relatively high accuracy in estimating the horizontal attitude angles, the gap between them became more obvious. The yaw angles of both the SO(3) and SE(3) models were corrected to incorrect values. The yaw angle error of the SO(3) model was about $150^{\prime }$, whereas the SE(3) model exhibited an even larger error of $210^{\prime }$. Despite the challenges encountered, both models presented in this paper demonstrated stable correction towards the true value. Specifically, the ECI-SE(3)-L model displayed a yaw angle error of $10.10^{\prime }$, and that of the ECI-SE(3)-R model is $8.40^{\prime }$. The results are demonstrated in Figure 3. Analysis of the state covariance in this test revealed that the SE(3) model experienced a rapid decline in the variance of the yaw angle error at the initial stage, leading to an erroneous value, as illustrated in Figure 4. To mitigate this issue, we adjusted the observation variance to regulate the filtering convergence rate of the models, allowing the filters to converge gradually. As a result, the yaw angle error of the SE(3) model was reduced to approximately $30.48^{\prime }$. Under the same observation variance, the ECI-SE(3)-L and the ECI-SE(3)-R models were still capable of accurately estimating the yaw angle, with errors of $12.85^{\prime }$ and $11.69^{\prime }$, respectively. However, the estimation accuracy of the SO(3) model was further impaired. The attitude angle error curves of the four models after the observation variance adjustment are shown in Figure 5.

The above comparisons indicate that the proposed models exhibit excellent attitude estimation performance in the turntable experiment, especially in the case of large initial misalignment angles and strong maneuvers. The filters based on the traditional SE(3) model converge too early, leading to the overreliance on inaccurate state estimates in the initial filtering stage and neglecting new observations, resulting in a notable degradation of estimation performance and the insensitivity to sudden observation errors or system changes. In contrast, the proposed models expand the dimensionality of the state model, but distribute errors more explicitly and precisely, demonstrating better robustness under inaccurate system models. With regard to the stability of the models in the early stage of the filtering, the SE(3) model exhibits significant fluctuations and overshoot, while the ECI-SE(3)-R model consistently exhibits good stability. Futhermore, the similar results obtained from the left- and right-invariant error models corroborate the earlier statement of their equivalence.

### 4.2. Car-Mounted Field Experiment

**Figure 6:**
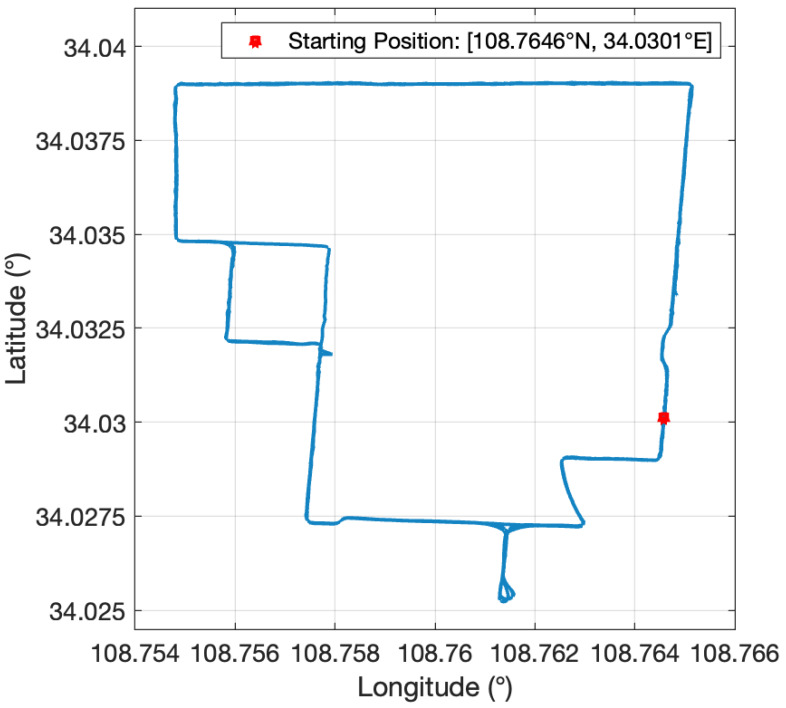
The trajectory of the car-mounted field experiment.

In this subsection, a set of car-mounted experimental data were processed to further validate the superiority of the proposed models when using low-precision inertial sensors. The experiment employed a Micro-Electro-Mechanical System (MEMS) SINS, specifically the STIM300 model. The triaxial gyroscope of the SINS has a drift rate of about 0.3°/h and a random noise intensity of 0.15°/$\sqrt{h}$, and the triaxial accelerometer bias is about 50 μg. The velocity accuracy of the GNSS used is about 0.1 m/s, and its position accuracy is 1 m. The SINS output frequency is 125 Hz, and the GNSS is recorded at 1 Hz. The main parameters of the sensors are listed in Table 2. The total duration of the experiment was approximately 3000 s, and the vehicle’s trajectory is shown in Figure 6. Additionally, an inertial-level fiber optic SINS was placed in the car, and its computational attitude was designated as the reference for comparison.

**Figure 7:**
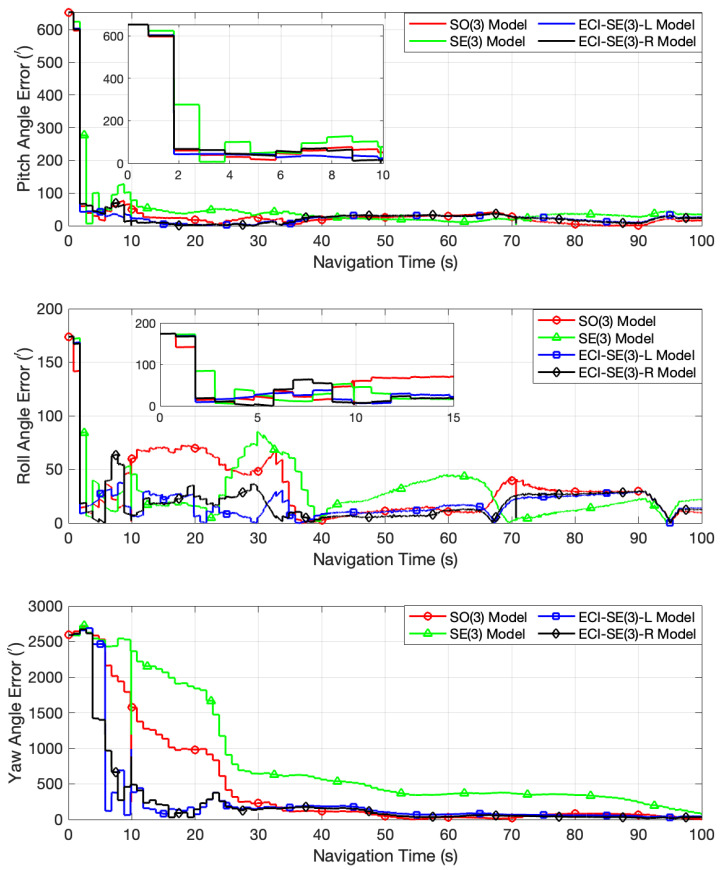
The attitude angle error curves of the four models with 0–100 s range in the car-mounted field experiment.

**Figure 8:**
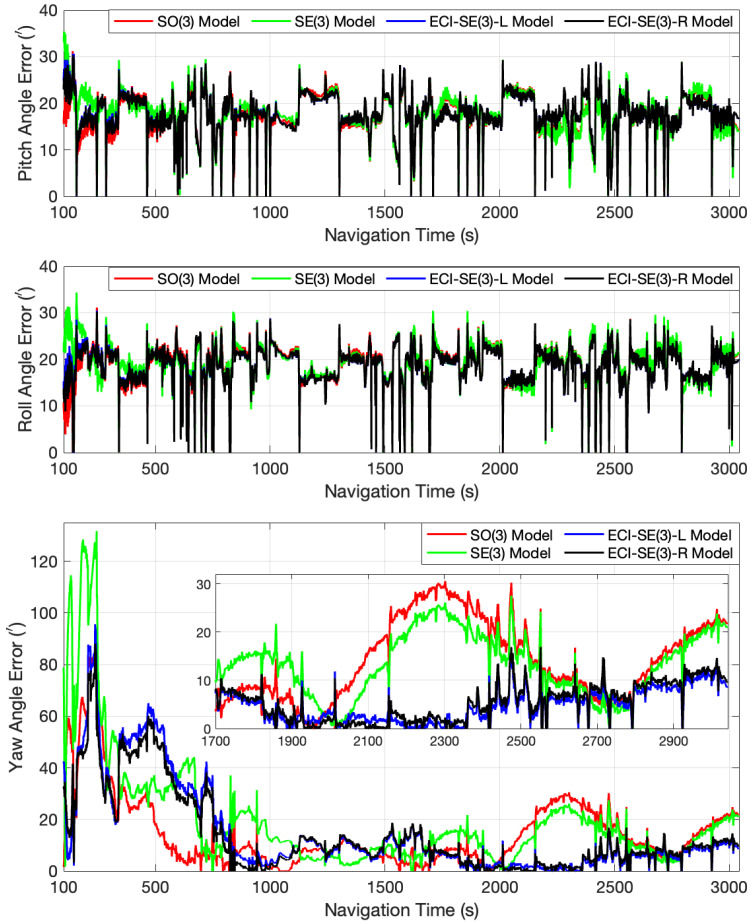
The attitude angle error curves of the four models after 100 s in the car-mounted field experiment.

Due to the fact that the attitude estimation accuracy of the four models is similar when the initial misalignment angle is not significant, the initial misalignment angles were set to [$10^{\circ }$, $10^{\circ }$, $50^{\circ }$] to evaluate the models’ performance. Figure 7 and Figure 8 depict the error curves for the pitch, roll, and yaw angles computed by the four models within the 0–100 s range and after 100 s, respectively. These curves illustrate that both models proposed in this paper effectively rectify the initially substantial inaccuracies in the three attitude angles, bringing them close to the true values within a relatively brief timeframe, and subsequently maintaining stable estimation accuracy. The conventional SO(3) model exhibits acceptable efficacy in correcting pitch angle discrepancies but demonstrates considerable oscillations in the early stage when addressing roll angle errors. Moreover, its rate of correcting yaw angle errors is considerably slower compared to the proposed models. Consistent with the findings of the preceding turntable experiment, the SE(3) model demonstrates heightened variability during the initial filtering phase and necessitates a longer duration to accurately adjust the yaw angle to approximate the true value, which is probably attributed to the imprecise estimation of common frame errors, causing the deterioration in the calibration attitude angles instead. After correcting the attitude angle errors to around zero, the vehicle attains a state of stable motion, resulting in the comparable estimation accuracy of the four models for the two horizontal attitude angles, as evidenced by the significant overlap in their error curves. When considering the estimation of the yaw angle within the time range of 300–700 s, the proposed models exhibit lower accuracy compared to the other two models. However, beyond approximately 1700 s, the estimation accuracy of the proposed models reliably surpasses that of the other two.

Table 3 and Table 4 present the Mean Absolute Error (MAE) and Root Mean Square Error (RMSE) values for the three attitude angles of the four models, (marking the pitch, roll, and yaw angles as θ, γ, and ψ), specifically within the time intervals of 0–100 s and after 100 s, respectively. The advantages of the proposed models in effectively addressing significant initial misalignment angles are evident. Additionally, when the vehicle remains in a stable state, the proposed models are capable of maintaining an equivalent level of accuracy to that of the traditional models.

### 4.3. Discussion

The results of two experiments using SINS with different accuracies demonstrate that the proposed models significantly improve attitude accuracy compared to traditional models under large initial misalignment angles and high dynamic maneuvers. The primary reasons for this improvement include:

Firstly, traditional methods often employ first-order Taylor expansion to approximate the SINS error equations, resulting in substantial errors in scenarios with large initial misalignment angles. In contrast, the proposed models inherit the logarithmic linear properties of the Lie group and its Lie algebra, enabling the transformation of the nonlinear error equations in the Lie group space into linear equations in the Lie algebraic space.

Secondly, the error propagation equations in traditional methods are not independent of global states, meaning that global parameters with large errors can lead to incorrect estimations of error parameters, which then feed back into the global states. This dependency requires traditional methods to spend more time correcting to the true value or even failing to do so when the initial misalignment angle is large. The method proposed in this paper is quasi-globally independent. Referring to Equations (14)–(16), (18), (28)–(30) and (32), the error propagation equations of the *b*-frame are independent of the global attitude and velocity, allowing both attitude and velocity errors to propagate independently. However, according to Equations (21)–(23), (25), (35)–(37) and (39), the error propagation equations of the *n*-frame are not globally independent, but they are only related to global velocity and position. This is inherently determined by the definition of the *n*-frame, which is based on the current position and the reference ellipsoid coordinate system. Fortunately, in most integrated navigation systems, velocity and position can be accurately estimated in a relatively short time, which will not pose a serious impact on the error propagation of the *n*-frame.

Thirdly, in the turntable experiment, despite repeated large and fast swaying maneuvers, the proposed method accurately tracks the attitude after the turntable returned to stationary, outperforming traditional methods. This accuracy is partly due to the quasi-global independence of the proposed models. Additionally, the Lie group-based SINS models constructed in this paper accurately compensate for the cone errors and sculling errors generated during large maneuvers through the relationship between the Lie group and its Lie algebra, as shown in Equation (11).

## 5. Conclusions

This paper develops the kinematic models of the SINS and its errors in the Earth-Centered Inertial coordinate system under the SE(3) group algorithm framework. With the Earth-Centered Inertial coordinate system as the reference, both the changes in the local geographic coordinate system and the body coordinate system and their errors are divided into two independent processes, so as to separate and decouple the factors and error terms that affect them. Additionally, the co-representation of attitude and velocity based on the SE(3) group takes the common frame errors into account. Through a turntable experiment and a car-mounted field experiment, the effectiveness of the proposed models in improving attitude estimation accuracy was demonstrated, particularly for the situation with strong maneuverability and large misalignment angles, and it was shown that the proposed models have better stability and robustness compared to the conventional SE(3) group models. The mathematical model of SINS studied in this paper holds broad applicability in precision navigation contexts such as missile guidance, geophysical measurement, and various civil applications like unmanned vehicle attitude determination and human gait analysis. However, the current study primarily explores the joint representation of attitude and velocity in the same Lie space, while the propagation of position and its errors remains in the general Euclidean space. Future research directions may involve extending the proposed models to accommodate alternative attitude representation methods like quaternion and Rodrigues parameters and investigating analogous models within the ${\mathrm{SE}}_{2}\left(3\right)$ group. Such endeavors hold promise for further advancing the understanding and practical implementation of robust navigation systems across diverse operational scenarios.

## Figures and Tables

**Table 1:** Main parameters of the sensors in the turntable experiment.

SINS	Gyroscope	Drift Rate	0.005°/h
		Random Noise	0.001°/$\sqrt{h}$
	Accelerometer	Bias	50 μg
GNSS	Position	Random Noise	10 m
	Velocity	Random Noise	0.1 m/s

**Table 2:** Main parameters of the sensors in the car-mounted field experiment.

MEMS-SINS	Gyroscope	Drift Rate	0.3°/h
		Random Noise	0.15°/$\sqrt{h}$
	Accelerometer	Bias	50 μg
GNSS	Position	Random Noise	1 m
	Velocity	Random Noise	0.1 m/s

**Table 3:** The MAE and RMSE of the attitude angle errors of the four models within 0–100 s range in the car-mounted field experiment.

	MAE (′)			RMSE (′)		
	θ	γ	ψ	θ	γ	ψ
SO(3) Model	32.67	31.53	454.58	87.67	41.55	862.84
SE(3) Model	48.18	29.49	843.43	98.27	40.30	1160.52
ECI-SE(3)-L Model	32.36	19.30	267.14	87.57	29.49	640.89
ECI-SE(3)-R Model	32.77	19.99	242.92	87.97	30.72	579.00

**Table 4:** The MAE and RMSE of the attitude angle errors of the four models after 100 s in the car-mounted field experiment.

	MAE (′)			RMSE (′)		
	θ	γ	ψ	θ	γ	ψ
SO(3) Model	17.43	18.98	14.73	17.96	19.42	20.79
SE(3) Model	17.93	19.33	19.81	18.46	19.75	29.64
ECI-SE(3)-L Model	17.80	18.81	14.65	18.25	19.17	22.95
ECI-SE(3)-R Model	17.75	18.76	13.92	18.20	19.12	20.99

## Data Availability

The datasets analyzed in the Turntable Experiment section of the current study are not publicly available but are available from the corresponding author on reasonable request, and the datasets analyzed in the car-mounted Field Experiment section of the current study are available in http://www.psins.org.cn/dhsj (accessed on 9 June 2024).

## Abbreviations

The following abbreviations are used in this manuscript:

SINS	Strapdown Inertial Navigation System
GNSS	Global Navigation Satellite System
DVL	Doppler Velocity Log
BCH	Baker-Campbell-Haussdorff
ECI	Earth-Centered Inertial frame
ECEF	Earth-Centered Earth-Fixed
IEKF	Invariant Extended Kalman Filter
KF	Kalman Filter
MAE	Mean Absolute Error
RMSE	Root Mean Square Error
